# Investigating the Inter-Tube Conduction Mechanism in Polycarbonate Nanocomposites Prepared with Conductive Polymer-Coated Carbon Nanotubes

**DOI:** 10.1186/s11671-015-1191-x

**Published:** 2015-12-16

**Authors:** Isaac Aguilar Ventura, Jian Zhou, Gilles Lubineau

**Affiliations:** Physical Sciences and Engineering Division, COHMAS Laboratory, King Abdullah University of Science and Technology (KAUST), Thuwal, 23955-6900 Saudi Arabia

**Keywords:** Polymers, Spectroscopy, Electrical properties, Carbon nanotubes, Nanocomposites

## Abstract

A well-known strategy to improve the electrical conductivity of polymers is to dope them with high-aspect-ratio and conductive nanoparticles such as carbon nanotubes (CNTs). However, these nanocomposites also exhibit undesirable properties such as damage-sensitive and history-dependent conductivity because their macroscopic electrical conductivity is largely determined by the tunneling effect at the tube/tube interface. To reduce these issues, new nanocomposites have been developed with CNTs that have been coated with a conductive layer of poly(3,4-ethylenedioxythiophene)poly(styrenesulfonate) (PEDOT/PSS). It has been posited that the insulating region between the CNTs is replaced by a conductive polymer bridge; this has not been proven up to now. We propose here to investigate in-depth how the macroscopic conductivity of these materials is changing when (1) varying the frequency of the electrical loading (impedance spectroscopy), (2) varying the mechanical hydrostatic pressure, and (3) varying the voltage of the electrical loading. The response is systematically compared to the one of conventional carbon nanotube/polycarbonate (CNT/PC) nanocomposites so we can clarify how efficiently the tunneling effect is suppressed from these composites. The objective is to elucidate further the mechanism for conduction in such material formulations.

## Background

A carbon nanotube/polymer nanocomposite can be described as a percolated network of conductive fillers embedded in an insulating matrix. Its macroscopic electrical conductivity is mainly dominated by the inter-particle junction behavior [1].

In direct current (DC) conditions, the junction behavior has been explained by the tunneling effect [2] which described how charge carriers travel through the insulating barrier (typically 0.5 to 2 nm thick) from one electrode to another. With this model, the junction can be represented as a resistive element, the resistance of which depends on strain, temperature, and voltage [3].

However, analysis of nanocomposites in the frequency-dependent domain has revealed that the AC conductivity of conductive particle-filled nanocomposites follows the universal AC behavior of disordered solids [4–6]. In general, this signifies that the real part of the AC complex conductivity increases with frequency following a classical power law [3, 7, 8]. Experimental observation of the AC behavior from dielectric and resistive frameworks have led to the description of an equivalent electrical circuit in which the inter-particle junctions are represented as Voigt elements (combination of a resistive and a capacitive part in parallel configuration) [5, 9]. The frequency dependency of the purely resistive part has been explained in terms of the characteristic percolated network microstructure. While in DC the charge carriers must travel along the percolated path throughout the sample, in AC, they only travel a probed distance which is inversely proportional to the frequency. As the probe distance is smaller with increasing frequency, many charge carriers travel mostly within favorable paths such as low-resistance junctions and nanoparticle bundles while highly resistive junctions are avoided [5, 7–11]. On the other hand, the capacitive element arises from the dielectric properties of the resin separating the conductive electrodes (the carbon nanotubes (CNTs)). Thus, under the influence of an AC, the charges accumulate on the junction and create polarization effects [12]. The frequency dependency of this element has also been described by works measuring the dielectric constant [4, 12, 13].

Recently, we have investigated the electrical and thermal properties and piezoresistive behavior of multiwall carbon nanotubes (MWCNTs)/polycarbonate (PC) nanocomposites in which the MWCNTs have been coated with the conductive polymer poly(3,4-ethylenedioxythiophene)poly(styrenesulfonate) (PEDOT/PSS) via a solution method [14, 15]. Our observations suggest that the MWCNTs are responsible for the construction of the percolated network, but the charge carrier transport is done through the PEDOT/PSS coatings.

The objective of this study is to clarify how the charge transfer mechanism is affected by the presence of the PEDOT/PSS coating at the inter-tube junctions. In particular, we observe the frequency dependency of the resistive and capacitive parts by performing a single electrical impedance spectroscopy experiment. We also present the results of the effect of hydrostatic pressure on conductivity and the voltage-current behavior. By comparing the electrical responses of this material with the expected features of classical nanocomposites, we can get some information about the efficiency of the PEDOT/PSS coating in replacing the insulating resin at the tube/tube interface.

## Methods

**Fig. 1:**
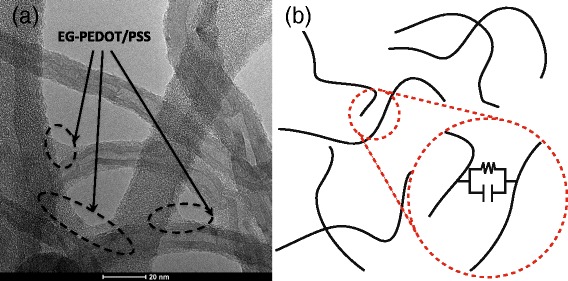
Morphology of the conductive filler percolated network. **a** TEM image of EPP-coated MWCNTs, highlighting the EPP coating and inter-connections. **b** Equivalent electrical circuit representation of the conductive filler percolated network. The junctions are represented by Voigt elements

We previously described [14, 15] the processing and the morphological characterization of CNT/PC and PEDOT/PSS-coated CNT/PC nanocomposites (E-CNT/PC). First, a highly conductive PEDOT/PSS, denoted as EPP, is prepared by blending a PEDOT/PSS aqueous dispersion (Clevios PH1000, HC Starck) with 5 wt. % of ethylene glycol. Secondly, we coat the CNTs by exfoliating them in the EPP solution. The EPP:CNT weight ratio is 1.3:1, providing a coating thickness of approximately 10 nm [14]. Figure 1a shows a TEM image of stand-alone EPP-coated MWCNTs. This blend is subsequently incorporated into PC according to the route described in [14]. In the following study, we investigate both CNT/PC and E-CNT/PC formulations containing 1, 1.5, and 2 wt. % MWCNTs, based on the PC content.

Rectangular samples (60 mm ± 0.5 × 10 mm ± 0.5 × 300 *μ*m ± 30) are prepared by casting and hot pressing for E-CNT/PC and CNT/PC nanocomposites [14] and equipping with silver paint electrodes (gauge length: 25 mm ± 0.5). Thin film samples of EPP (rectangular samples: 30 mm ± 0.5 × 6 mm ± 1 × 40 *μ*m ± 0.5) were also prepared by spin-casting process to investigate the response of the PEDOT/PSS coating itself, which has never been reported. These were also equipped with silver paint electrodes separated by 19 mm.

We systematically investigated the change in electrical conductivity when these formulations are subjected to (1) AC frequency sweep, (2) hydrostatic pressure sweep, and (3) DC voltage sweep.

For the electrical impedance spectroscopy (EIS), the resistance and reactance of the samples were measured simultaneously with an Agilent E4980A Precision LCR meter in a four-probe configuration using Kelvin clips. The frequency range spans from 20 Hz to 2 MHz. The AC electrical conductivity and the tan *δ* were calculated accordingly.

For hydrostatic pressure testing, the samples were placed inside an air pressurized container in which the pressure could be regulated from 1 to 20 bar while the resistance of the samples was measured using a four-probe technique with an Agilent 1252B multimeter. Finally, the electrical conductivity, *σ*, with respect to electric field, *E*, was obtained from V-I curves using a Keithley 4200 semiconductor parameter analyzer and a Cascade probe station.

## Results and Discussion

**Fig. 2:**
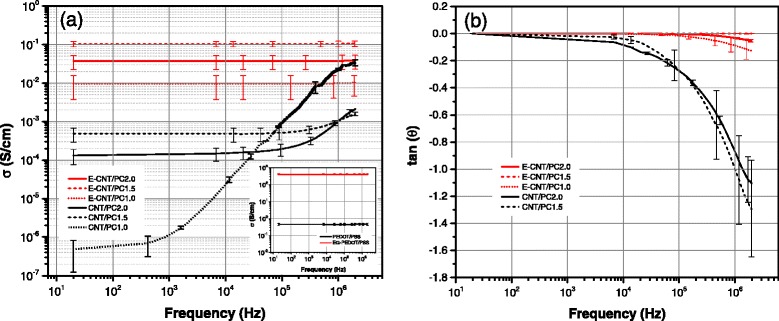
Electrical impedance spectroscopy results for the different formulations. **a** AC conductivity and **b** tan *θ* vs frequency for PC nanocomposites (and PEDOT/PSS films in the *insert*). The conductivity levels for PC nanocomposites shown are comparable to the ones previously reported [15]. The difference corresponds to expected anisotropy from the manufacturing process

Figure 2 summarizes the EIS results. The AC conductivity, *σ*(*ω*), is calculated as *σ*(*ω*)=*l*/*R*(*ω*)*A*. *R*(*ω*) is the real part of the impedance (*Z*(*ω*)=*R*(*ω*)−*iX*(*ω*)); *l* and *A* are the length and cross sectional area of the sample, respectively; and *X*(*ω*) is the reactance. While CNT/PC samples show the typical frequency-dependent response of conductive filler/polymer nanocomposites [6, 8], the E-CNT/PC samples’ response is frequency-independent (Fig. 2a). The EIS results of EPP (Fig. 2a, inset) also testify to a frequency-independent behavior. These observations suggest that in E-CNT/PC samples, the conduction mechanism is dominated by the purely resistive behavior of EPP. This becomes even more clear when looking at the difference in phase response, *θ*, between samples (Fig. 2b). Recall tan *θ*, defined as tan *θ*=*X*/*R*, represents the magnitude of the imaginary part (capacitive reactance) with respect to the resistive part. It can be observed that, in CNT/PC samples, the capacitive part largely increases with frequency. For samples such as CNT/PC1.5 and CNT/PC2.0, the capacitive part at low frequencies is insignificant with respect to the resistive part, but at high frequencies (>1 Mhz), the magnitudes of *R* and *X* are comparable to each other. This comes from the thin layer of dielectric PC as the inter-tube interface that acts locally as a capacitor. In contrast, the resistive behavior dominates in E-CNT/PC samples even at high frequencies with a negligible capacitive part. This behavior suggests that no polarization effects take place at the inter-tube junctions of E-CNT/PC samples.

**Fig. 3:**
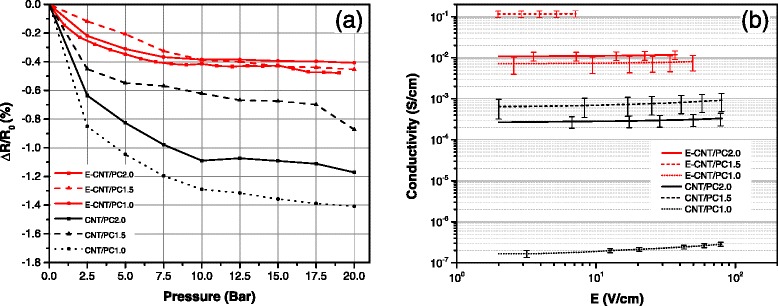
Change in electrical resistance with pressure and electric field strength. **a** Change in DC electrical resistance vs pressure and **b** electrical conductivity vs electric field strength

The results from the hydrostatic pressure test are shown in Fig. 3a. Because this test is carried out on DC conditions, it is expected to reveal information about the resistive element within the inter-particle junction. The purpose of the experiment is to submit the samples into a hydrostatic stress state. As the pressure rises, the inter-tube separation distance decreases which causes a decrease of the macroscopic resistance for the CNT/PC samples. This effect is limited due to the relatively low pressure applied, but it is noticeable especially for the low-concentration CNT/PC sample where the distance between nanotubes is initially greater. Most importantly, it clearly reveals a different behavior between both types of samples. In comparison to CNT/PC, E-CNT/PC samples feature a very stable macroscopic resistance, even at low-CNT concentration. This is consistent with the hypothesis that charge transfer is mainly ensured by the EPP coating both on the nanoparticles and at the tube junctions.

Finally, Fig. 3b shows the change in conductivity of PC nanocomposites with respect to the applied DC electric field. Polymer nanocomposites usually display a non-ohmic behavior [16, 17] which is a consequence of the non-linear effective resistance of the tunneling junctions [2]. As expected, this effect is noticeable in our CNT/PC samples in which inter-tube tunneling is the driving effect. This effect gets very attenuated in E-CNT composites. This is a first indication that tunneling is replaced by less voltage-dependant mechanisms in this new formulation.

## Conclusions

The results of the presented characterization techniques indicated that the presence of the EPP coating on the MWCNTs changes dramatically the electrical behavior of the percolated network of CNTs at the level of the inter-tube junctions. In CNT/PC samples, the junction behavior is dependant on the distance between fillers. It can be represented by a Voigt element (a combination between a resistive and a capacitive element), albeit the resistance and the capacitance are themselves frequency dependent as the probing distance becomes smaller with increasing frequency. On the other hand, in E-CNT/PC samples, the junctions are replaced by a conductive polymer bridge with a purely resistive behavior. The existence of this bridge is here fully demonstrated by its impact of the macroscopic response to voltage sweep, frequency sweep, and pressure sweep.
